# Peptide-Ligand Binding Modeling of siRNA with Cell-Penetrating Peptides

**DOI:** 10.1155/2014/257040

**Published:** 2014-07-24

**Authors:** Alfonso T. García-Sosa, Indrek Tulp, Kent Langel, Ülo Langel

**Affiliations:** ^1^Institute of Chemistry, University of Tartu, Ravila 14a, 50411 Tartu, Estonia; ^2^Institute of Technology, University of Tartu, Nooruse 1, 50411 Tartu, Estonia; ^3^Department of Neurochemistry, Stockholm University, 106 91 Stockholm, Sweden

## Abstract

The binding affinity of a series of cell-penetrating peptides (CPP) was modeled through docking and making use of the number of intermolecular hydrogen bonds, lipophilic contacts, and the number of sp3 molecular orbital hybridization carbons. The new ranking of the peptides is consistent with the experimentally determined efficiency in the downregulation of luciferase activity, which includes the peptides' ability to bind and deliver the siRNA into the cell. The predicted structures of the complexes of peptides to siRNA were stable throughout 10 ns long, explicit water molecular dynamics simulations. The stability and binding affinity of peptide-siRNA complexes was related to the sidechains and modifications of the CPPs, with the stearyl and quinoline groups improving affinity and stability. The reranking of the peptides docked to siRNA, together with explicit water molecular dynamics simulations, appears to be well suited to describe and predict the interaction of CPPs with siRNA.

## 1. Introduction

Cell-penetrating peptides (CPPs) are short (generally five to 40 amino acid) peptide sequences that are able to deliver biologically active cargos into the cell cytoplasm and nucleus by means of their ability to cross cell membranes [1, 2]. Molecules of particular interest for delivery across membranes are drugs and nucleic acids, such as small interfering ribonucleic acids (siRNA), given that this allows the normally inactive siRNA access to bind to a cell's specific nucleotide sequence that performs a given task, such as regulating endogenous genes [3]. To better develop silencing gene technology and its associated benefits, a better understanding of the mechanism in which CPPs bind to genetic material and help introduce it into cells is needed. This would also provide suggestions on how to design peptides with better efficiency.

Positively charged (basic) groups on amino acids like lysine and arginine provide features that are helpful for binding to siRNA. CPPs can bind covalently or noncovalently to siRNA. Arginine-rich motifs, zinc fingers, RNA recognition motifs, small molecules, and tethered approaches, among others, have been used to bind RNA [4]. Recent tools can help in profiling peptide and chemical compounds in their binding and delivery, such as ligand efficiency indices [5–11], fraction of sp3 orbital hybridization carbons [12], as well as the atomic binding interactions between peptide-ligand, including explicit water [13], and dynamic effects [13]. The guanidino group on an arginine residue is especially valuable in binding nucleic acids, given that it can perform electrostatic, hydrogen bond, cation-*π*, and *π*-*π* interactions. Artificial neural networks and principal components analysis have been employed to study cell-penetrating peptides in an attempt to classify them according to their permeability [14]. Boltzmannian stochastics have also been used to calculate populations of 3D structures of CPPs using PepLook, calculating both intra- and intermolecular interactions [15]. Molecular dynamics simulations have also been carried out on penetratin and the TAT peptide with lipid bilayers [16–18], as well as of dimer peptides [19] or zinc-fingers [20] with DNA, and the CPP CADY in complex with siRNA [21]. Molecular modeling can also discover ligands to nucleic acids [22]. Some CPPs have been developed to improve their load delivery, such as in the case of NF51, PF3, PF6, and TP10 [23–26]. Recently determined X-ray crystal structures of siRNA in complex with peptides provide structural information about their binding. Docking coupled with molecular dynamics simulations can provide clues on the structural, energetic, and dynamic effects of CPP to nucleic acid binding. Binding partner atoms and functional groups, their conformational rearrangements and persistence over time are part of these clues, which in turn allow proposing suggestions for further modification of peptides for increased affinity and/or specificity to particular nucleotide sequences.

## 2. Methods

### 2.1. Modeling

The structure of the double-stranded 21 nucleotide-luciferase siRNA (luc-siRNA) was generated using Maestro version 9.2 [27], using as a template the structure of Tav2b/siRNA complex from the Protein Data Bank [28] structure file 2ZI0. The template has the closest crystal structure to luc-siRNA with 8 matching base pairs, as well as helical peptides (Tomato aspermy virus 2b (TAV2b) proteins) bound to the siRNA. This structure allows using a bound (*holo*) conformation of the siRNA, in order to improve docking calculations to the receptor with respect to those using an* apo* or unbound structure. Mismatching nucleotides were mutated and the resulting structure (in complex with two alpha-helical peptides in the major groove) was energy minimized using MacroModel version 9.9 [29], prior to conducting a 1.2 ns molecular dynamics simulation to relax and equilibrate the complex structure. The relaxed and minimized siRNA structure (sequence 5′-ACGCCAAAAACAUAAAGAAAG and antisense 5′-UUCUUUAUGUUUUUGGCGUCU) was then extracted from this complex for further use in docking the CPP peptides NF51, PF3, PF6, and TP10 [23–26].

### 2.2. Docking

Peptide structures were generated with Maestro v. 9.2 [27] and energy-minimized. The peptides were then docked flexibly (flexible ligands, rigid target) with GOLD v. 5.0.1 [30] using ChemScore [31], and employing the following conditions suited for flexible ligands: autoscale = 2; Population: popsiz = auto, select_pressure = auto, n_islands = auto, maxops = auto, niche_siz = auto; Genetic operators: pt_crosswt = auto, allele_mutatewt = auto, migratewt = auto; Flood fill: radius = 40 Å. The auto option allows adjusting the conformational sampling according to the number of rotatable bonds in the ligands, and this provides for the flexibility in the peptide ligands.

A modified score of S(hbond ext) + (1.35∗S(lipo)) [32] was further developed by incorporating the number of sp3 carbons and used to rerank the peptides. S(hbond ext) measures the intermolecular hydrogen bond contributions to binding, and S(lipo) is a lipophilic term that is calculated between nonpolar carbon, nonionic chlorine, bromine, and iodine, and nonaccepting sulphur atoms [29]. These terms are dependent on the distance between two atoms pairs and on how much they differ from ideal values for interaction. Sp3 carbons were calculated with Marvin Beans [33].

### 2.3. Downregulation Experiments

siRNA downregulation experiments were performed as previously reported [25, 26]. Briefly, in the case of TP10, PF3, and PF6, 100 *μ*M CPP stock solution was mixed with siRNA (10 *μ*M stock solution—the same siRNA sequence as used in the modeling) in MQ water in one-tenth of final treatment volume (i.e., 50 *μ*L), using molar ratio (MR) MR30 in serum free media or MR40 in serum experiments. Complexes were formed for 30 min at RT and added to HEK cells, grown to 60% confluence in a 24-well plate, in 450 mL growth media. After 4 h, 1 mL of fresh media was added to wells and cells were incubated for the indicated times. Further, the cells were lysed using 100 mL of 0.1% Triton X-100 in Hepes Krebbs Ringer buffer. After 30 min lysis on ice, luciferase expression was measured using the Promega Luciferase Kit on a 96-well Glomax luminometer (Promega).

In the case of NF51, EGFP-CHO cells were seeded in 24-well plates 24 h prior to experiments. siRNA was mixed with CPP at different molar ratios (MR 5–30) in MQ water. Complexes were formed and cells were treated as described above. After the indicated time, media were removed and the cells were rinsed with PBS, detached from the plate, and suspended with PBS containing 5% FBS, and FACS analysis was performed.

### 2.4. Molecular Dynamics

The program Desmond version 3.1 [34, 35] was used to perform molecular dynamics (MD) simulations. Structures were first energy-minimized in MacroModel version 9.9 [27] in an implicit water environment using the OPLS2005 force field and GB/SA continuum solvation model with 2000 steps of Conjugate-Gradient and Steepest Descent, or until a gradient threshold of 0.5 kcal/mol/Å was reached. Further salt (Na^+^ and Cl^−^) ions were inserted to neutralize the system until reaching a 0.15 M concentration. Complex structures were then solvated using TIP3P [36] water molecules in a periodic orthorhombic box of 8 Å added to each direction extending from the solute, to give total dimensions of 73.4 × 48.8 × 80.3 Å. Simulations were conducted with a constant temperature of 300 K using a Nosé-Hoover chain thermostat [37] and Martyna-Tobias-Klein barostat methods [38]. A timestep of 1 fs was employed, and simulations were run for 10 ns in total. The first 1 ns were used as equilibration time. The particle mesh Ewald algorithm was used for calculating long-range electrostatic interactions [39]. Trajectories were further analyzed with Desmond.

In addition, the peptide-siRNA complexes were modeled in explicit water and with an excess of 40 peptides to one siRNA molecule.

## 3. Results and Discussion

The CPPs studied have sequences that are shown in Table 1.

**Figure 1:**
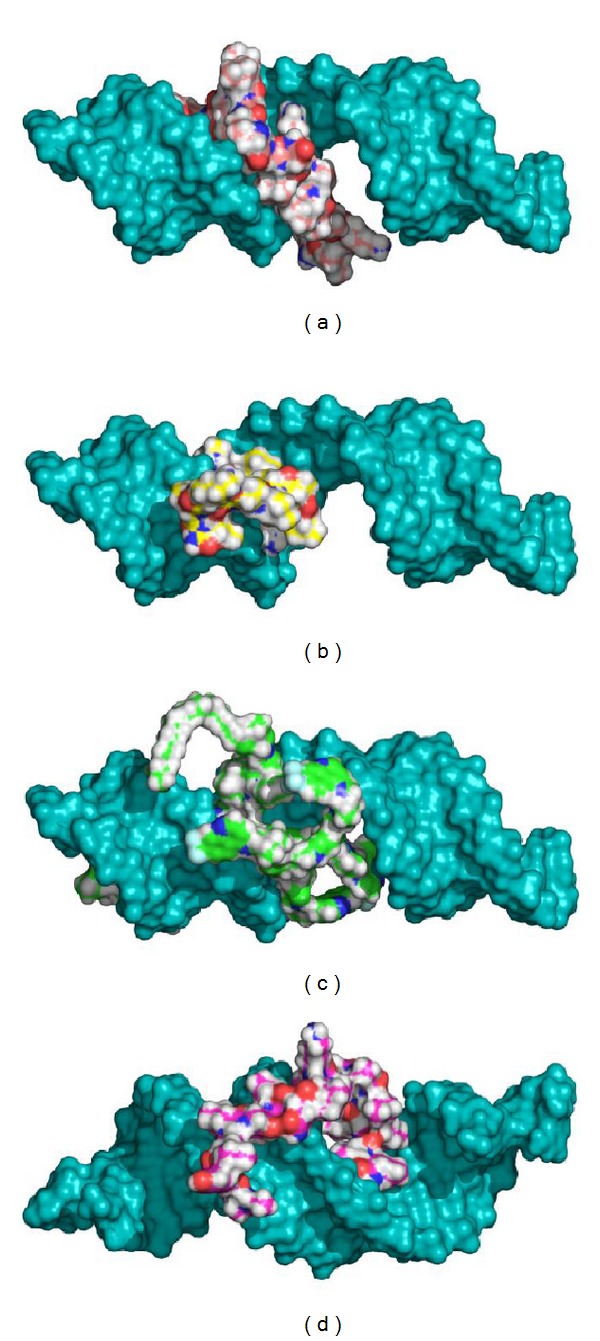
Docked binding modes for the complexes of siRNA and cell-penetrating peptides: (a) NF51, (b) PF3, (c) PF6, and (d) TP10.

GOLD docked the peptides using a genetic algorithm that explores different conformations and positions of the peptide structures in the biomolecular target. After docking, the structures of the docked ligands appear to be well attached to the siRNA double-helix, making extensive use of interactions between the peptide and the major groove of siRNA. Hydrogen bonding and electrostatic, as well as lipophilic interactions, are dominant, as shown by their binding poses and scores. The binding conformations are such that the peptides prefer them to retain their secondary structure they had prior to binding, that is, a nine residue alpha-helix. The binding modes and ligand poses for the best complexes are shown in Figure 1. The best complexes were those with the deepest binding energy, and with the most realistic binding mode and plausible intermolecular interactions, and were chosen from at least four different independent runs.

The ranking of the peptides docked to the siRNA is shown in Table 2. The external hydrogen bonding term and the lipophilic term components of the ChemScore scoring function, as well as the total number of sp3 carbons in the molecule, were used and recomposed to create a modified score, called SusiScore. This modified score was composed as SusiScore = Number of sp3 carbons∗(S(hbond ext) + 1.35∗(S(lipo))). SusiScore makes use of the most important components of the ChemScore scoring function that have been correlated to binding affinity [31], as well as the sp3 carbons in the molecule, which have been correlated to the complexity and solubility of a compound [12], in order to create a new scoring and ranking procedure.

The calculation results are comparable to the experimentally determined efficiency of the different peptides of delivery of siRNA into cells, which are shown in Table 3 [25, 26]. There is the same trend between the downregulation experiments and the binding scores.

The reason for the better binding with siRNA for NF51, PF6, and PF3, as compared to TP10, would appear to be the stearyl tail present in the former three, but lacking in TP10. This lipophilic group acts as an anchor which fixes the peptide to the siRNA, providing a means for the peptide structure to stretch and embrace the majority of the siRNA helix. In addition, the quinoline groups of PF6 interact with the nucleic acid basepairs, giving the complex PF6*·*siRNA added stability.

An apparent explanation for the ability of the different peptides to deliver the siRNA into the interior of the cells may be the strength of their binding complexes. If, as speculated, the peptides can form pores in the cell membrane, a stronger binding to the siRNA would allow the peptide to drag the siRNA with it as it transverses the membrane. Peptides with a lower binding affinity would probably be able only to form pores, and the lower delivery of siRNA would be due to it only entering the cell through diffusion through the pores in the membrane created by the peptide. Stronger binding peptides may prolong the interaction with the siRNA and therefore accompany it through the pores in the cell membrane it has created.

The lipophilic stearyl tail that provided stronger binding for NF51, PF3, and PF6 with respect to TP10 may also play a role in the insertion into the lipid bilayer of the cell membrane, providing a better interaction with the lipophilic groups inside the lipid bilayer, resulting in likely easier formation of pores in the cell membrane, as well as binding interactions with siRNA.

A mechanism for CPP delivery includes the association in clusters with glycosaminoglycans on the cell membrane surface [40]. It seems likely that the CPP complexes with nucleic acids are then internalized into the cell through endocytosis [25] and even suggested being pinocytosis [18]. Given the stronger binding and stronger downregulation provided by NF51 and PF6 as compared to PF3 and TP10, as shown in the present study and in biological experiments, it also adds support to the thesis that the binding between CPPs and nucleic acids (at least for those peptides considered here) is not released until inside the cell. Perhaps their release occurs by localization in the lysozyme [41], given that the lower pH there allows for acid or ionic competition for the binding of the nucleic acid and disrupts the binding complex CPP*·*nucleic acid.

MD simulations of single CPPs bound to siRNA showed that the bound structures of peptides and siRNA obtained from docking were stable. The simulation quality analysis from Desmond showed stable and regular values for total energy, potential energy, pressure, and temperature (standard deviation lower than 2 K for all cases).

**Figure 2:**
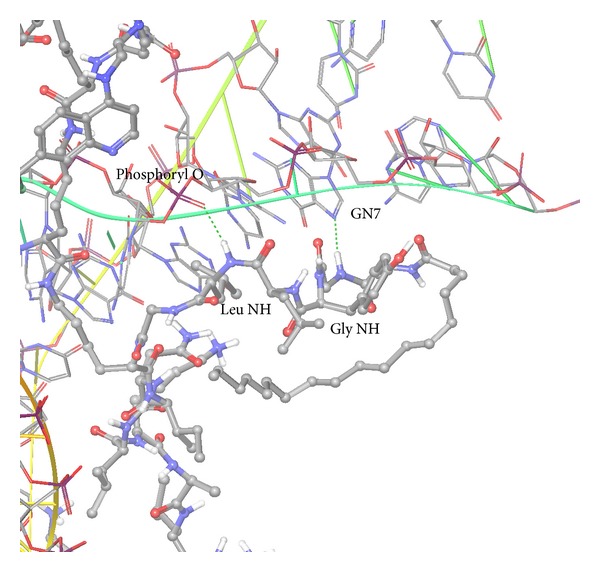
Molecular dynamics snapshot structure of the CPP*·*siRNA complex for PF6. Peptide is shown in ball and stick, siRNA in wireframe and green and yellow ribbons. Hydrogen bonds between the tail of PF6 and siRNA are shown in green dashed lines, from left: Leu NH → Phosphoryl O and Gly NH → Guanine N7. For clarity, explicit water molecules are not shown.

The complexes did not fall apart and remained strongly bound throughout the 10 ns simulations. The stearyl tail of PF6 bound to siRNA folded more closely to the siRNA as the simulation progressed. The secondary structure of the siRNA was stable and maintained its double-helix throughout all of the simulations. The RMSDs of the peptide backbone atoms were 1 Å for NF51, 1.5 Å for PF6, 1.5 Å for PF3, and 2 Å for TP10. The RMSDs of the siRNA backbone atoms were 2 Å for NF51, 1.5 Å for PF6, 2.5 Å for PF3, and 4.5 Å for TP10. The RMSDs of the siRNA and peptide backbone atoms together were 2 Å for NF51, 4 Å for PF6, 4 Å for PF3, and 4.5 Å for TP10.  Figure 2 shows a close-up view of the structure of the complex between the peptide PF6 and siRNA.

The MD simulation of TP10 bound to siRNA showed a higher RMSD for peptide and siRNA compared to the other CPPs, probably related to the weaker binding affinity for the complex. The RMSDs of the peptide sidechains and modification groups were small and ranged from 1.5 Å for the K_3_QN_4_ group in PF6, 0.5 Å for the stearyl group in PF6, 0.75 Å for the stearyl group in PF3, and 1.2 Å for the stearyl group in NF51. These values indicate the stability of the complex for siRNA bound to NF51, and PF6, and to a smaller extent PF3, and even less stable was the complex with TP10.

During the simulation of the PF6 complex, the stearylated tail of the peptide approached siRNA from an extended into a folded conformation, forming a lid over the other residues and bases by generating two stable intermolecular hydrogen bonds: the peptide glycine (the “first” glycine, i.e., that closest to the stearyl tail) backbone NH donating a hydrogen bond to the siRNA guanine (G) N7 (distance shortened to 2.9 Å) and between the peptide leucine backbone NH donating a hydrogen bond to the phosphoryl (nonester) oxygen in the siRNA backbone (distance varied from over 5 to 2.6 Å). The former hydrogen bond is specific to certain siRNA bases, specifically guanine and adenine, and by modifying this specific amino acid in the peptide (such as modifying the peptide backbone to a group less susceptible to hydrolysis or without a hydrogen bond donor), or modifying the guanine or adenine nucleotides in this position in the siRNA, binding can be enhanced or suppressed leading to specific binding affinity for designed peptides and siRNA sequences. The latter hydrogen bond, on the other hand, given that it occurs between the peptide and the backbone of the siRNA, is less specific and would be expected to be conserved in different siRNA sequences. These hydrogen bonds and contacts between peptide and nucleic acid give suggestions for further modification of the peptides and nucleotide sequences for tuning of specificity and binding affinity.

**Figure 3:**
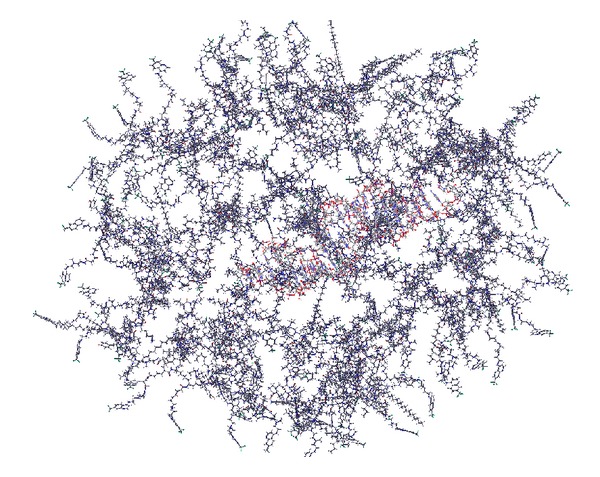
40 peptide units of PF6 surrounding siRNA.

The simulation of 40 CPPs and siRNA in a ball showed that the structures were stable over 1 ns. The mechanism of binding allows one CPP peptide to interact strongly in the main groove, while the subsequent peptides have less specific and less strong interactions with the charged backbone, arranged in subsequent binding shells, similar to solvation shells. The structure of this ball for PF6 in complex with siRNA can be seen in Figure 3. The ball simulations showed the structures to be stable, where the constructs of PF6 showed a longer stability than TP10, whose structure fell apart before 1 ns.

Given the docking rescores and the molecular dynamics trajectories, which show stability for the peptides, and the rankings corresponding to experiment, the importance of appropriate sidechains and modifications for the CPPs is demonstrated in the improved binding affinity for siRNA for PF6 and NF51, as well as greater stability for the complex in MD simulations, and perhaps an improved ability to cross the cell membrane. In addition, the sidechains and modifications can be further optimized to improve delivery, downregulation of activity, and safety, and for the quinoline groups such as those in PF6, to possibly improve selectivity for particular nucleic acid sequences. The procedure developed in the present work may be used to design modifications to the CPP core and sidechains or extending groups and follow or predict their interactions with siRNA.

## 4. Conclusions

The newly proposed reranking developed in the present study, SusiScore, together with flexible ligand docking and explicit water molecular dynamics simulations appear to be able to describe the interactions of cell-penetrating peptides with siRNA. The same guidelines that rule the binding of small molecules to receptors are present in peptide binding. In addition, the present study shows that the number of sp3 carbons improve the ranking of calculated results. Therefore, the use of the number of sp3 carbon atoms that has been shown to be important in small molecule binding would also be appropriate for peptide binding. The stearyl group present in PF3, PF6, and NF51, and the quinoline group present in PF6, increase the binding affinity and stability of their complexes with siRNA compared to TP10, which lacks these groups. Further modifications to the core, sidechains, and extending groups of CPPs and other cell-penetrating compounds may be designed and their interactions modeled through the procedure described here. The impact of the different cores, sidechains, and extending groups will be seen in binding affinity to siRNA, as well as solubility, stability, and efficiency in delivering and downregulating nucleic acids.

## Figures and Tables

**Table 1:** Amino acid sequences for cell-penetrating peptides.

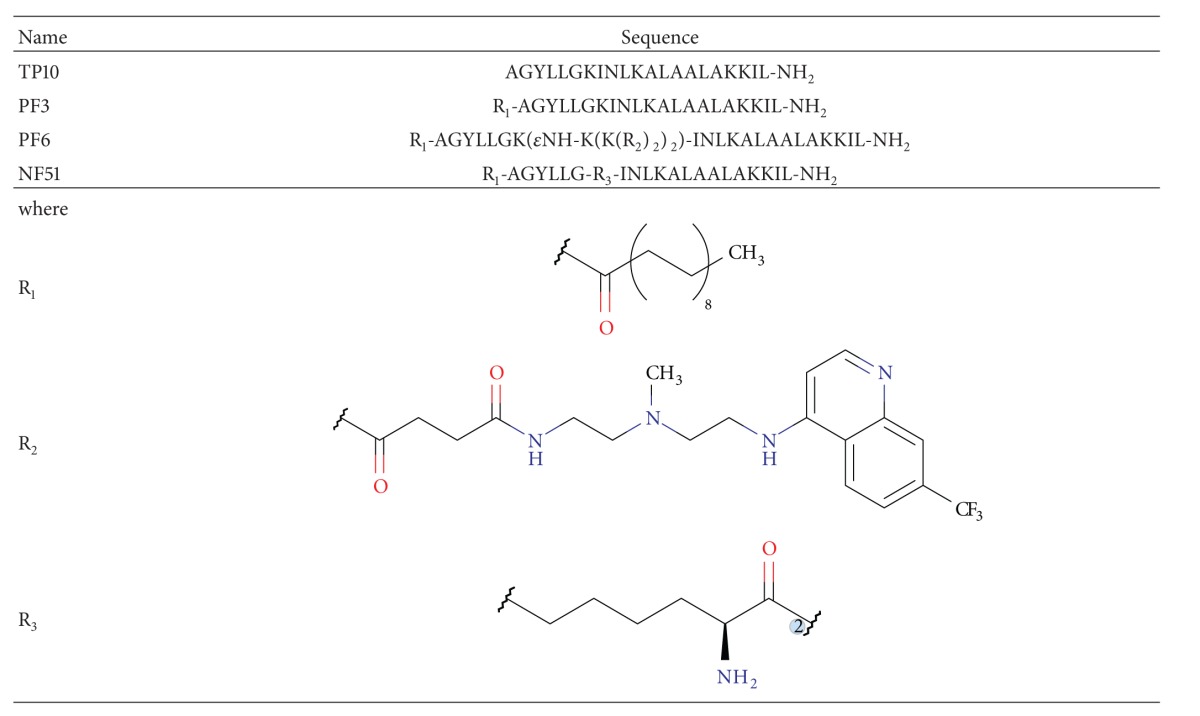

**Table 2:** Docking score components, modified scores, and ranks of peptides bound to siRNA.

Rank	CPP	*S*(hbond ext)	*S*(lipo)	Modified score from GOLD	sp3 carbons	SusiScore
1	PF6	3.33	133.38	183.39	144	29,592
2	NF51	2.92	150.06	205.50	93	17,055
3	PF3	3.59	128.84	177.52	93	16,509
4	TP10	4.88	72.97	103.39	77	7,961

**Table 3:** siRNA downregulation % in HEK cells using luc-siRNA (5′-ACGCCAAAAACAUAAAGAAAG and antisense 5′-UUCUUUAUGUUUUUGGCGUCU).

CPP	Serum containing media
PF6 (MR40)	80%
NF51 (MR10)	70%
PF3	0%
TP10	0%

## Abbreviations

CPP: Cell-penetrating peptide
siRNA: Small interfering ribonucleic acids
MQ water: Milli-Q water
HEK cells: Human embryonic kidney cells
RT: Room temperature
EGFP-CHO cells: Enhanced green fluorescent protein-Chinese hamster ovary cells
PBS: Phosphate-buffered saline
FBS: Fetal bovine serum
FACS: Fluorescence-activated cell sorting
MD: Molecular dynamics
MR: Molar ratio.
